# Editorial: Recurrence of liver tumors: the issue of iterative approaches

**DOI:** 10.3389/fonc.2023.1201092

**Published:** 2023-06-14

**Authors:** Michele Finotti, Alessandra Bertacco, Francesco Enrico D’amico, Alessandro Vitale, Enrico Gringeri, Umberto Cillo

**Affiliations:** ^1^ Annette C. and Harold C. Simmons Transplant Institute, Baylor University Medical Center, Dallas, TX, United States; ^2^ 4th Surgery Unit, Regional Hospital Treviso, Dipartimento di Scienze Chirurgiche Oncologiche e Gastroenterologiche (DiSCOG), University of Padua, Padua, Italy; ^3^ Dipartimento di Scienze Chirurgiche Oncologiche e Gastroenterologiche (DiSCOG), General Surgery 2-Hepato-Pancreato-Biliary Surgery and Liver Transplantation Unit, University of Padua, Padua, Italy

**Keywords:** hepatocellular carcinoma, colorectal liver metastases (CLM), cholangiocarcinoma, liver transplant (LT), iterative approach

Primary and secondary liver tumors are mainly treated with a multimodal treatment (chemotherapy and liver surgery) aiming for the best long-term patient Overall Survival (OS). However, Liver Tumor Recurrence (LTR) is frequent, and in this context, multiple tools are now available: liver resection with parenchyma sparring techniques, mini-invasive approaches, and multiple and multimodal surgical and medical therapies, based on the liver tumor’s features.

In this Frontiers issue, “*Recurrence of Liver Tumors: The Issue of Iterative Approaches*”, we analyzed the role of surgery on the LTR treatment, through 16 peer-reviewed open-access publications including 173 authors and experts in the field from 8 different countries, 2 study groups, European Hepatocellular Cancer Liver Transplant (EurHeCaLT) and Italian Liver Cancer (ITA.LI.CA) group and 39 reviewers and co-editors.

The first set of papers is dedicated to hepatocellular carcinoma (HCC), the most common primary liver cancer and the second most common cause of cancer-related death worldwide. HCC is mostly associated with cirrhosis/end-stage liver disease and, compared to other liver tumors, the prognosis is linked not only to the tumor features but also to the stage of the underlying liver disease.

Liver transplant (LT) is the best therapeutic option but, mostly due to organ shortage, is not always feasible.

In this setting, the iterative approach is the natural consequence of the HCC history after any type of treatment: the intrahepatic recurrence. Even with first-line treatments, LT, and curative hepatectomy, intrahepatic recurrence is common, as shown by Lai et al.

If HCC recurrence is expected, indications, timing, and type of treatment according to the patient’s stratification by the risk of recurrence (personalized medicine) are still a matter of debate.

In the first series of articles, we acknowledge that different therapeutic options are accessible (repeat LR, ablation techniques, TACE) in a setting of iterative treatment and we discussed the lacking of high evidence-based studies on the management of recurrent HCC.

The mini-invasive approach is the key to iterative treatment for RLT, especially in HCC. The advantages, compared to open surgery, are fewer intra and postoperative complications, faster postoperative recovery, and possible repetition of the treatment in a shorter time.

Chen et al. evaluated the role of curative liver resection for recurrent HCC with a mini-invasive approach compared to open resection with a propensity score–based study and confirmed by an up-to-date meta-analysis.

The study showed that laparoscopic liver resection is associated with lower blood loss, better post-operative liver function, and shorter post-operative course with comparable operative time, complication, and mortality rate confirmed also by Hao et al.

Thanks to the improvement of laparoscopic surgical techniques, the study showed that the mini-invasive approach can be safely applied also in challenging tumors, such as recurrent HCC located in posterosuperior segments or with a maximum size of >5 cm. The authors showed also an interesting relationship between lower post-operative inflammation-based markers and enhanced recovery in the population treated with the mini-invasive approach.

Mini invasiveness and fast recovery are not the only essential element for the iterative process, but also sparing as much as liver possible for possible future treatments, particularly in patients with liver cirrhosis and/or multiple nodules/portal hypertension.

Cheng et al. addressed this issue, showing that liver resection with very narrow surgical margins (<1mm) has outcomes comparable to those with wider margins.

These initial results seem promising, but long-term oncologic outcomes and future randomized trials are needed. Furthermore, the role of the robotic approach in the iterative treatment of HCC recurrence is an ongoing topic that has to be addressed and evaluated as well.

As shown, liver resection is the first line treatment of HCC recurrence when LT is not indicated/possible. However, repeat hepatectomy is not always feasible, due to liver function, number/size of HCC recurrence, and/or patient performance status.

Recent studies showed that Trans Arterial ChemoEmbolization (TACE) is a possible but, if applied alone, limited alternative in terms of efficacy.

Pelizzaro et al. showed that TACE can be an important iterative tool for an upward shift toward curative therapies that provide higher survival benefits compared to TACE repetition (LR, LT, and ablation).

Also, Zheng et al. showed that TACE in combination with radiofrequency ablation (RFA) (TACE-RFA) has similar outcomes compared to repeat hepatectomy in the treatment of recurrent HCC in terms of safety, overall survival, and progression-free survival. To note, for the first time the study showed similar results for HCC diameter > 5cm and lower post-operative complications compared to repeated liver resection. Considering the low impact TACE-RFA has on the postoperative course of the patient, this tool can be considered as a possible alternative to liver resection and/or as a bridge to LT. Further studies are mandatory.

In the second series, we proposed studies evaluating the impact of HCC recurrence after LT.

In a large European cohort, Lai et al. confirmed LT as the best therapeutic option for HCC with the excellent long-term outcome at 5 and 10 years, despite the recent widened of HCC selection criteria outside Milan criteria.

The study pointed out that HCC recurrence can happen >5 years after LT, especially in patients with previous multimodal iterative treatment, as described by Pagano et al. Adequate post-LT surveillance and further iterative treatment even after LT has to be considered.

Therefore, in the case of HCC recurrence, multiple therapeutic options can be offered in different possible combinations and timing (re-resection + TACE, TACE + RFA, re-resection + RFA, etc.), underlining the importance of a mini-invasive approach and liver sparing to enhance faster recover and reduce the impact on liver dysfunction.

However, there is still debate about the timing and type of tool to offer and which population would benefit the most.

We addressed this issue in the third series of articles, proposing evidence-based tools to predict accurately the pre- and post-operative HCC recurrence and to guide the most beneficial pharmacological or surgical treatment/monitoring for that specific patient (personalized medicine).

Different staging systems can be used to clinically stage and guide the HCC treatment, such as the American Joint Commission on Cancer (AJCC), the Barcelona Clinic Liver Cancer (BCLC) system, the Cancer of the Liver Italian Program (CLIP) system, the albumin-bilirubin (ALBI) grading system. However, they are not designed to guide physicians on the treatment of recurrent HCC, and one of the limitations of the current known risk features for early recurrence is that most of them are based on postoperative histopathological tissue.

Recently, the China liver cancer (CNLC), an evidence-based staging system, gained interest thanks to its ability to perform globally better than other systems, especially compared to BCLC. Liao et al. proposed two nomograms to implement the CNLC for recurrent HCC with three independent risk factors for OS (cirrhosis, GGT, and tumor differentiation) and one for RFS (AFP). This resulted in a better ability to predict survival and HCC recurrence in patients treated with curative hepatectomy, helping the physician to identify a high-risk population with potential early recurrence.

With the same goal, He et al. proposed to use of multimodal (MRI/CT) radiomics models to predict HCC prognosis and recurrence before treatment, and Chen et al. proposed two nomograms incorporating the most important predictive factor for recurrence and OS (microvascular invasion).

Knowing a specific population with a higher risk of recurrence can help to guide toward a more aggressive or iterative treatment (i.e. LR + TACE, LR + RFA).

Furthermore, Zou et al. proposed a novel blood index signature (BIS) able to accurately predict HBV-associated HCC (HBV-HCC) recurrence after curative hepatectomy. Based on the risk of HCC recurrence, the study identified a high-risk group that benefits specifically from adjuvant TACE.

In the second set of papers, we translated the same concept of iterative surgery to all the tumors with a high risk of recurrence after primary treatment, such as ColoRectal Liver Metastases (CRLM) and cholangiocarcinoma (CCA), concepts summarized by Aquina et al. and Bekki et al. in their comprehensive literature reviews.

As well HCC, CRLM, and CCA face the issue of a lack of patient stratification and an accurate prognostic model able to predict the recurrence and guide the application of an iterative approach. Jin et al. faced the issue of patient stratification in recurrent CRLM proposing a nomogram based on age, TN stage, neoadjuvant chemotherapy, and primary tumor position to identify optimal patients that may benefit the most from an iterative treatment.

On the same line, Liu et al. developed a prognostic model with good calibration for risk estimation of CCA recurrence.

Liver resection is the treatment of choice, but ablation techniques are often applied as iterative CRLMs treatment, thanks to the mini-invasive approach (percutaneous or video-assisted) and liver tissue sparing. Guadagni et al. showed that for CRLMs <4cm, liver microwave ablation has mid-term oncological outcomes similar to liver resection. In case of multiple lesions, and recurrent and deep liver segments, ablation can be an effective alternative to resection to improve liver sparing and expedite the post-operative course, given possible future further treatments.

In conclusion, in tumors with a high risk of recurrence, the iterative approach is essential to improve the patient’s OS and keep the liver disease under control. Multiple therapeutic sessions with different tools are often required to achieve a complete liver tumor regression and radicality. Keys elements for the iterative approach are mini-invasiveness, low post-operative patient impact, fast recovery, and repeatable over time.

The series pointed out that the decision of the therapeutic options should be tailored to that specific patient (personalized medicine) based on the type of liver cancer and the patient’s features. The efficacy of that specific surgical tool (resection, ablation, TACE, etc.) is not merely related to the technique itself but it is mainly associated with the correct patient selection that will benefit the most from that specific procedure. Further studies evaluating the best sequence and timing of the iterative surgery, and implementing the current staging system are necessary to achieve the personalized medicine concept.

## Author contributions

All authors listed have made a substantial, direct, and intellectual contribution to the work and approved it for publication.

